# Effects of Elevated CO_2_ and Nitrogen Deposition on Ecosystem Carbon Fluxes on the Sanjiang Plain Wetland in Northeast China

**DOI:** 10.1371/journal.pone.0066563

**Published:** 2013-06-20

**Authors:** Jianbo Wang, Tingcheng Zhu, Hongwei Ni, Haixiu Zhong, Xiaoling Fu, Jifeng Wang

**Affiliations:** 1 Key Laboratory for Vegetation Ecology, Ministry of Education, Institute of Grassland Science, Northeast Normal University, Changchun, Jilin Province, P. R. China; 2 Institute of Natural Resources and Ecology, Heilongjiang Academy of Sciences, Harbin, Heilongjiang Province, P.R. China; DOE Pacific Northwest National Laboratory, United States of America

## Abstract

**Background:**

Increasing atmospheric CO_2_ and nitrogen (N) deposition across the globe may affect ecosystem CO_2_ exchanges and ecosystem carbon cycles. Additionally, it remains unknown how increased N deposition and N addition will alter the effects of elevated CO_2_ on wetland ecosystem carbon fluxes.

**Methodology/Principal Findings:**

Beginning in 2010, a paired, nested manipulative experimental design was used in a temperate wetland of northeastern China. The primary factor was elevated CO_2_, accomplished using Open Top Chambers, and N supplied as NH_4_NO_3_ was the secondary factor. Gross primary productivity (GPP) was higher than ecosystem respiration (ER), leading to net carbon uptake (measured by net ecosystem CO_2_ exchange, or NEE) in all four treatments over the growing season. However, their magnitude had interannual variations, which coincided with air temperature in the early growing season, with the soil temperature and with the vegetation cover. Elevated CO_2_ significantly enhanced GPP and ER but overall reduced NEE because the stimulation caused by the elevated CO_2_ had a greater impact on ER than on GPP. The addition of N stimulated ecosystem C fluxes in both years and ameliorated the negative impact of elevated CO_2_ on NEE.

**Conclusion/Significance:**

In this ecosystem, future elevated CO_2_ may favor carbon sequestration when coupled with increasing nitrogen deposition.

## Introduction

Atmospheric CO_2_ concentrations are predicted to double by the end of the century [1]. Increasing CO_2_ concentrations usually stimulate ecosystem C uptake through photosynthesis [2], [3]; however, concurrent increase in ecosystem respiration due to increasing root respiration and enhanced priming of soil organic matter may lead to ecosystem carbon losses [4], [5]. The difference between photosynthetic and respiratory fluxes is called net ecosystem exchange (NEE) [6]. Thus, NEE depends on the relative changes in C uptake and release, but the extent of variance in C uptake and release in response to elevated CO_2_ is uncertain.

In addition, N level are expected to vary in response to global N enrichment caused by anthropogenic activities [7]. According to one estimate, by 2050, 200 Tg N yr^−1^ will be released and then deposited into the earth’s surface [8]. In general, N is a limiting factor for plant growth and net primary production. Consequently, it has been conjectured that N deposition could increase carbon dioxide uptake from the atmosphere [7], [9]. However, different ecosystem respiration responses to N addition have been reported in previous studies, including increases [10]–[12], decreases [13], [14] and no significant changes [15]. Therefore, it remains unclear whether N deposition leads to net ecosystem C sequestration [16], [17].

The progressive nitrogen limitation (PNL) hypothesis [18] suggests that N additions should enhance CO_2_ effects on plant productivity. Consequently, concurrent changes in atmospheric CO_2_ concentration and N deposition may potentially bring about complex interactive impacts on ecosystem functioning. There has been much research on the effects of elevated atmospheric CO_2_ concentrations and N deposition on the growth of individual plants, but relatively little research on the impact of ecosystem C fluxes on natural vegetation at ecosystem scale [4], [19]–[21]. In particular, there has been no detailed study evaluating the interactive effect of elevated CO_2_ and N addition on ecosystem C ﬂuxes on wetland ecosystems. Wetlands have globally significant stores of soil carbon and sink for CO_2_ [22], and consequently play an important role in the global carbon cycle.

A field experiment manipulating elevated CO_2_ and N deposition was conducted, beginning in May 2010, to examine the potential influences of climate change on a temperate wetland in northeast China. The objectives of this study are to address the following three questions: (1) How do elevated CO_2_ and N deposition impact GPP, ER and NEE? (2) Whether elevated CO_2_ and N deposition interactively affect ecosystem carbon flux on the Sanjiang Plain wetland? (3) How do environmental (e.g., soil temperature, soil water content) and biotic (e.g., vegetative cover) factors regulate the responses of the ecosystem carbon flux to elevated CO_2_ and N deposition?

## Materials and Methods

### Ethics Statement

All work was undertaken with relevant permissions from the Honghe National Nature Reserve, China for our observational and field studies. The field studies did not involve endangered or protected species. Data will be made available upon request.

### Study Site

This study was conducted at the Field Experimental Establishment of Institute of Natural Resources and Ecology––the Honghe National Nature Reserve (47°49′N, 133°40′E) on the Sanjiang Plain, a wetland located in the Heilongjiang Province of Northeastern China. A low floodplain was formed by the Heilongjiang River, the Songhuajiang River and the Wusulijiang River. Sanjiang Plain is approximately 1.04×104 km^2^ in area and is N deficient, as indicated by the strong stimulation of gross primary productivity after N addition [23]. Annual N deposition is 7.6 kg hm^−2^ in this region [24]. The area has a temperate continental monsoon climate. The mean annual temperature ranges from 1.4 to 2.9°C, with the average maximum of 21–22°C occurring in July and the average minimum −18°C in January. The mean annual precipitation is 500–650 mm and 80% of rainfall occurs in summer. The frost-free period is 120–140 days. The growing season is limited to late April to early October. Meadow soil is the main soil type with 20% of soil organic matter content and 0.5% of total N at a 0–10 cm soil depth. On the Sanjiang Plain wetland, the native vegetation is characterized by the dominance of the perennial species *Calamagrostis angustifolia,* as well as the presence of *Carex appendiculata*, *Carex lasiocarpa, Carex pseudocuraica, Glyceria spiculosa* and *Lathyrus quinqunervuius.*

### Open Top Chamber Design

Three pairs of Open Top Chambers (OTC), each 1.8 m high with an octagonal ground surface area of 10.78 m^2^, were set up in early May 2010. They were constructed with a 5-m-wide buffer zone between them to prevent cross contamination. Light intensity in each chamber was 95% of that in open space. The precipitation intensity was identical inside and outside of the chambers. We supplied carbon dioxide to the chambers through a pipe with pinholes connected to industrial CO_2_ tanks outside the chambers. We adjusted the CO_2_ supply in accordance with wind speed and CO_2_ concentrations by taking constant measurements with an infrared gas analyzer.

### Experimental Design

The experiment used a paired, nested design with elevated CO_2_ as the primary factor and nitrogen addition as the secondary factor. Three pairs of OTC were used, and within each pair one was randomly assigned to receive the elevated CO_2_ treatment (700 ppm, E) and the other as the control (370 ppm, C). The CO_2_ enrichment began in May 2010 and continued each year for the entire growing season (from May to September) during the daytime (6∶00 a.m. to 6∶00 p.m.). The CO_2_ supply was shut off during the night. At each elevated CO_2_ and control OTC chamber, there were two subplots, one control and the other N addition (N). To each N addition subplots, 8 g N m^−2^ yr^−1^ (N, in the form of liquid NH_4_NO_3_) were applied once a year in early May, while the controls were sprayed with distilled water only. Thus, there were four treatments, elevated [CO_2_] and enriched N deposition (EN), elevated [CO_2_] and ambient N deposition (E), ambient CO_2_ and enriched N deposition (N) and ambient CO_2_ and ambient N deposition as a control (C). Each treatment was replicated three times. The experiments lasted two growing seasons.

### Climate Data Soil Microclimate

Climate data were obtained from a Hobo meteorological station, which was approximately 250 m distant from the experimental plots. Soil temperature (T_soil_) at a 10 cm depth and soil water content (SWC) at a 0–10 cm soil depth were continuously recorded with an ECH_2_O dielectric aquameter (EM50/R Decagon Ltd, Pullman, WA, USA) at 2-h intervals from May 2010 to September 2011 (except when the sample plots flooded in August).

### Ecosystem-Level Gas Exchange Measurement

Ecosystem gas exchange was measured on 7 sampling dates during the 2010 and 2011 growing season, respectively (May to September, except when the sample plots flooded). Measurements were performed under cloudless conditions from 9∶00 a.m. to 11∶00 a.m. to avoid the high air temperature and humidity at noon. In May 2010, one square aluminum frame (0.5 m×0.5 m) was inserted 3 cm into the soil of each subplot, approximately 0.5 m away from the edge. The frames provided a ﬂat base between the soil surface and the CO_2_ sampling chamber. When ecosystem gas exchange was measured, we turned off the CO_2_ supply system at least 10 min prior so that the CO_2_ concentration was consistent in chamber. Ecosystem gas exchange was measured with a transparent chamber (0.5×0.5×1.0 m) attached to an infrared gas analyzer (IRGA; LI-6400, Li-Cor, Lincoln, NE, USA), which was placed on the frame of each subplot. Four small electric fans were running continuously to promote the mix of air within the chamber during the measurement. Twelve consecutive recordings of the CO_2_ concentration were taken at 10-s intervals during a 120-s period. The rates of CO_2_ fluxes were calculated from the time-courses of the concentrations to determine net ecosystem exchange (NEE). Using the approach of Steduto [25], we converted the concentration change to flux-per-unit of soil area. After taking the NEE measurements, the chamber was vented, replaced on each frame and covered by an opaque cloth. The ER was then measured. Gross primary productivity (GPP) was then determined by the sum of NEE and ER (GPP = NEE+ER). Positive NEE values refer to net uptake of C by the ecosystem, and negative NEE values represent a net loss of C from the ecosystem.

### Green Plant Cover

For each subplot, the percent cover estimate (percentage of ground area within a subplot covered by green vegetation) was taken. These plots were scored once each year in July.

### Leaf Gas Exchange Measurement

Leaf net photosynthetic rate in the dominant plant species *C. angustifolia* was measured with an open gas-exchange system (Li-6400; Li-Cor, Lincoln, NE, USA). In each subplot, three fully expanded leaves of *C. angustifolia* were selected and measured once each year in July. Leaf gas exchange was measured in the mornings between 9∶00 a.m. and 11∶00 a.m. on clear days. During the measurement, leaves were illuminated at 1500 µmol m^−2 ^s^−1^ using an LED light system.

### Statistical Analyses

The seasonal mean values used in this study were calculated from the monthly mean values, which were first averaged using all measurements from the same month. We examined soil microclimate (T_soil_ and soil water content) and ecosystem C fluxes (NEE, ER and GPP) results for the entire 2010–2011 period and used repeated measures of ANOVAs to test for main effects and interactions, and whether these changed over time (contrasting both the measuring date and year). The between-subject effects were evaluated as elevated CO_2_, N addition and their interactions, and within-subject-effects were measuring date, year and their interactions with elevated CO_2_, and N addition. Regression with correction for autocorrelation and stepwise multiple linear analyses were used to examine the relationships of ecosystem C fluxes with soil temperature, soil water content and vegetation cover in the two growing seasons. Statistical analyses were conducted with SPSS software (SPSS Inc, Chicago, IL, USA), and figures were plotted with SigmaPlot 11.0 software.

## Results

### Microclimate Changes Induced by Elevated CO_2_ and N Addition Treatments

**Figure 1:**
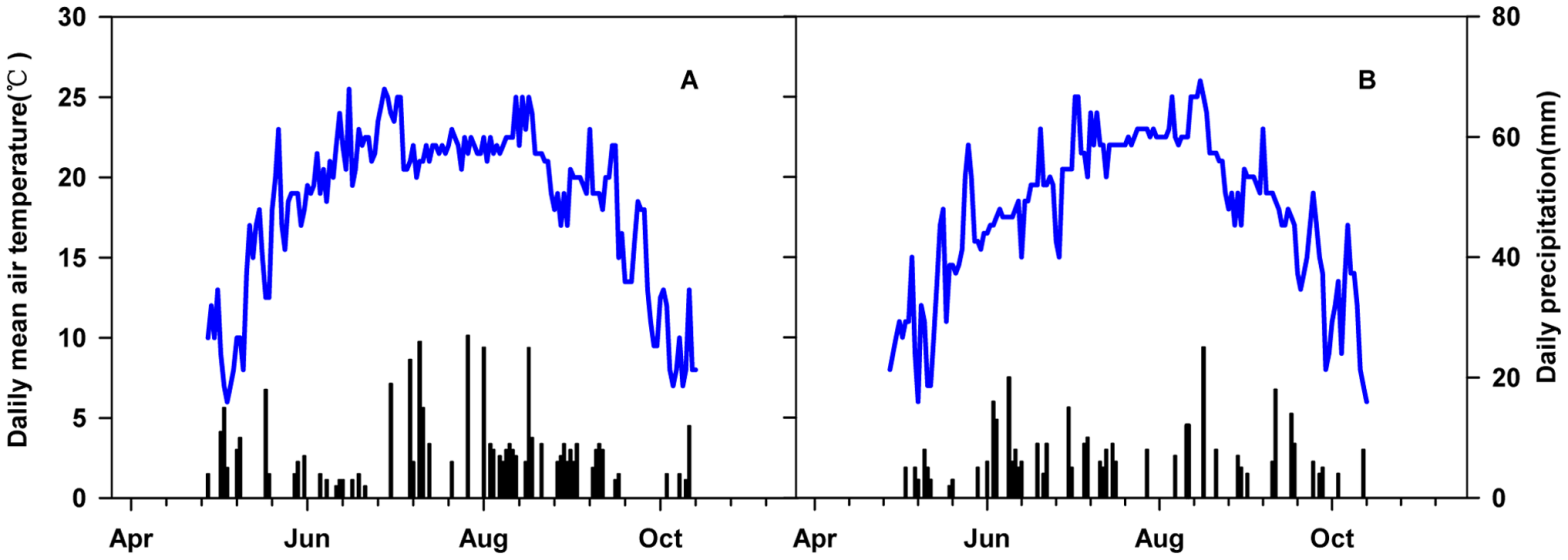
Daily mean air temperature (line) and daily precipitation (bars) in 2010 (A) and 2011(B) during the growing seasons. Data were obtained from a Hobo meteorological station approximately 250 m from the experimental plots.

In comparison with the long-term averages (1981–2010) of mean air temperature (MAT, 17.3°C) during the growing season, both 2010 (18.4°C) and 2011 (17.8°C) had higher MAT. However, during the growing season, 2010 had higher precipitation (495 mm) and 2011 had lower precipitation (364 mm) than the long-term mean precipitation (MP, 459 mm; Fig. 1). Furthermore, mean air temperature was obviously higher in 2010 than that in 2011 in the early growing season– the monthly mean temperature was higher by 0.95°C in May and 3.12°C in June (Fig. 1). Moreover, the average T_soil_ of 2010 was significantly higher than that of 2011 (P<0.001, Table 1, 2). N addition significantly increased T_soil_ but decreased soil water contents (SWC) (P<0.05). Soil water content was higher in the elevated CO_2_ plots than in the control plots (P<0.001, Table 2).

**Table 1:** Growing season means of soil temperature (T_soil_, °C), soil water content (SWC) and ecosystem C fluxes (NEE, ER and GPP, µmol m^−2^s^−1^) under different treatments in 2010 and 2011.

		T_soil_	SWC	NEE	ER	GPP
**2010**	C	16.12±0.2	66.4±0.7	7.67±0.05	9.80±0.22	17.47±0.55
	N	16.45±0.3	66.1±1.6	8.61±0.30	11.09±0.47	19.71±0.58
	E	16.02±0.2	68.3±0.5	6.64±0.14	11.96±0.40	18.61±0.45
	EN	16.82±0.2	67.3±0.8	8.64±0.30	13.27±0.41	21.91±0.71
**2011**	C	14.86±0.2	72.4±1.5	4.93±0.05	5.47±0.23	10.40±0.26
	N	15.18±0.2	71.4±2.5	5.72±0.21	5.67±0.31	11.39±0.52
	E	14.52±0.3	73.8±2.6	4.10±0.14	6.39±0.24	10.49±0.38
	EN	14.92±0.4	72.6±3.5	5.57±0.21	6.78±0.28	12.35±0.49

Values represent the Means± SE. C: control plots; N: N addition; E: elevated CO_2_; EN: combined elevated CO_2_ and N addition.

**Table 2:** Results (*P* value) of repeat-measurement ANOVA on the effects of year (Y), elevated CO_2_ (CO_2_), N addition (N), measuring date (D), and their interactions on soil temperature (T_soil_), soil water content (SWC), net ecosystem CO_2_ exchange (NEE), ecosystem respiration (ER) and gross primary productivity (GPP).

Effect	T_soil_	SWC	NEE	ER	GPP
D	<0.001	<0.001	<0.001	<0.001	<0.001
D×CO_2_	0.567	<0.001	<0.001	<0.001	0.048
D×N	0.003	0.015	<0.001	<0.001	<0.001
D×Y×CO_2_	0.038	<0.001	0.012	<0.001	<0.001
D×Y×N	0.709	0.071	<0.001	<0.001	0.016
D×CO_2_×N	0.796	0.001	0.04	0.002	0.052
D×Y×CO_2_×N	0.178	0.05	0.032	<0.001	0.145
Y	0.000	0.001	<0.001	<0.001	<0.001
CO_2_	0.655	<0.001	0.003	<0.001	0.007
N	0.022	0.014	<0.001	0.008	<0.001
N×CO_2_	0.447	0.598	0.007	0.851	0.196
Y×CO_2_	0.247	0.804	0.972	0.046	0.13
Y×N	0.592	0.514	0.244	0.078	0.079
Y×CO_2_×N	0.613	0.792	0.506	0.869	0.89

### Seasonal and Interannual Variations of Ecosystem CO_2_ Fluxes

**Figure 2:**
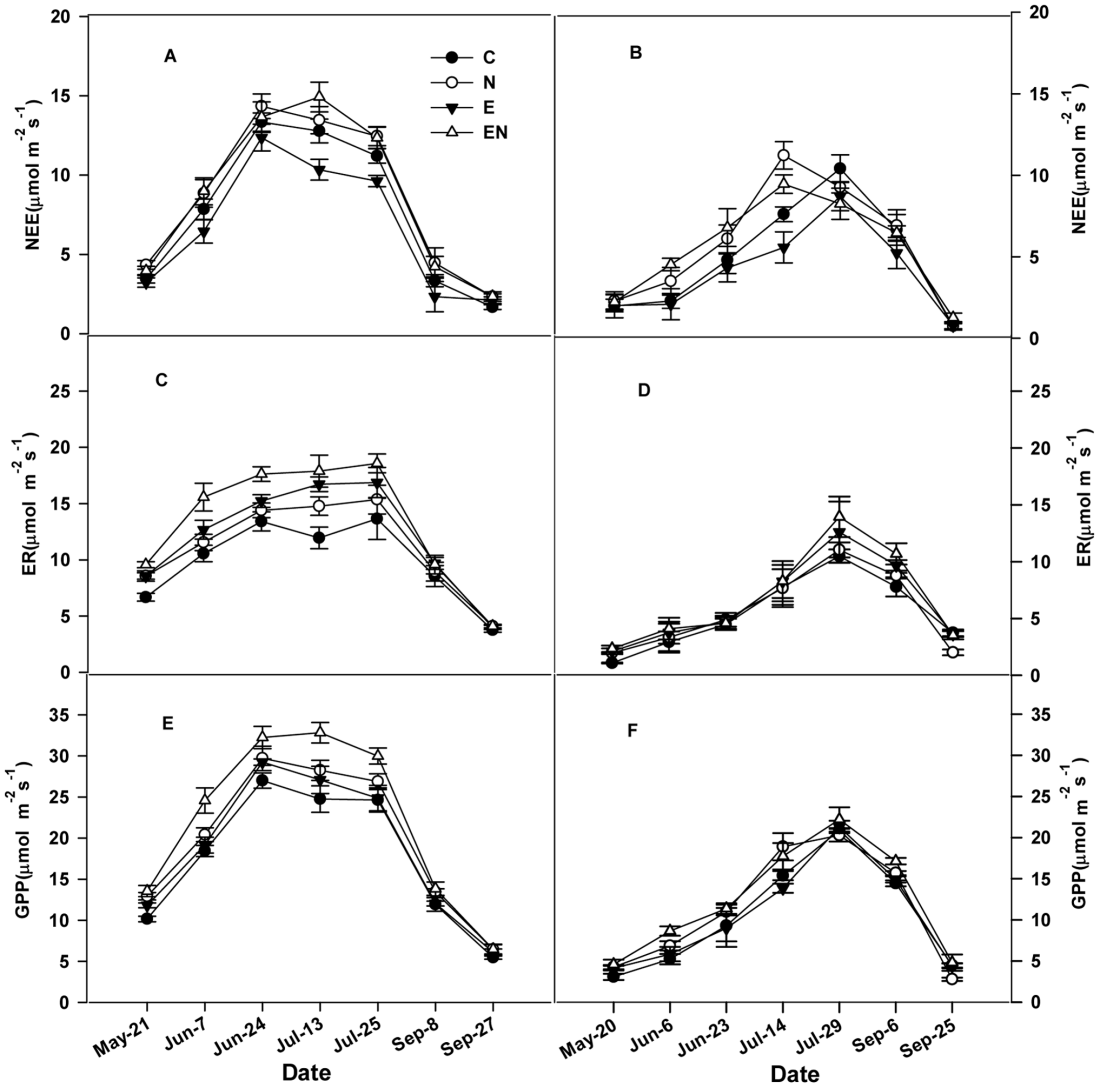
Seasonal dynamics of net ecosystem CO_2_ exchange (NEE); ecosystem respiration (ER) and gross primary productivity (GPP) in 2010 (left) and 2011 (right) (August values are missing because of flooding on the experiment plots). C: control plots; N: N addition; E: elevated CO_2_; EN: combined elevated CO_2_ and N addition.

The seasonal dynamics of ecosystem C exchange followed a parabolic-like pattern with higher values in summer and lower values in spring and autumn (Fig. 2), which was similar to the air temperature change. The rates of ecosystem CO_2_ flux (GPP, ER and NEE) were significantly lower in 2011 than in 2010 (Tables 1, 2; P<0.001). In the control plots, seasonal means for NEE, ER and GPP in 2010 were 56%, 79% and 68% greater than in 2011, respectively. The remarkable interannual variabilities in the ecosystem C fluxes might be partly attributed to differences in air temperature, especially in the early growing season.

### Effects of Elevated CO_2_ on Ecosystem C Fluxes

**Figure 3:**
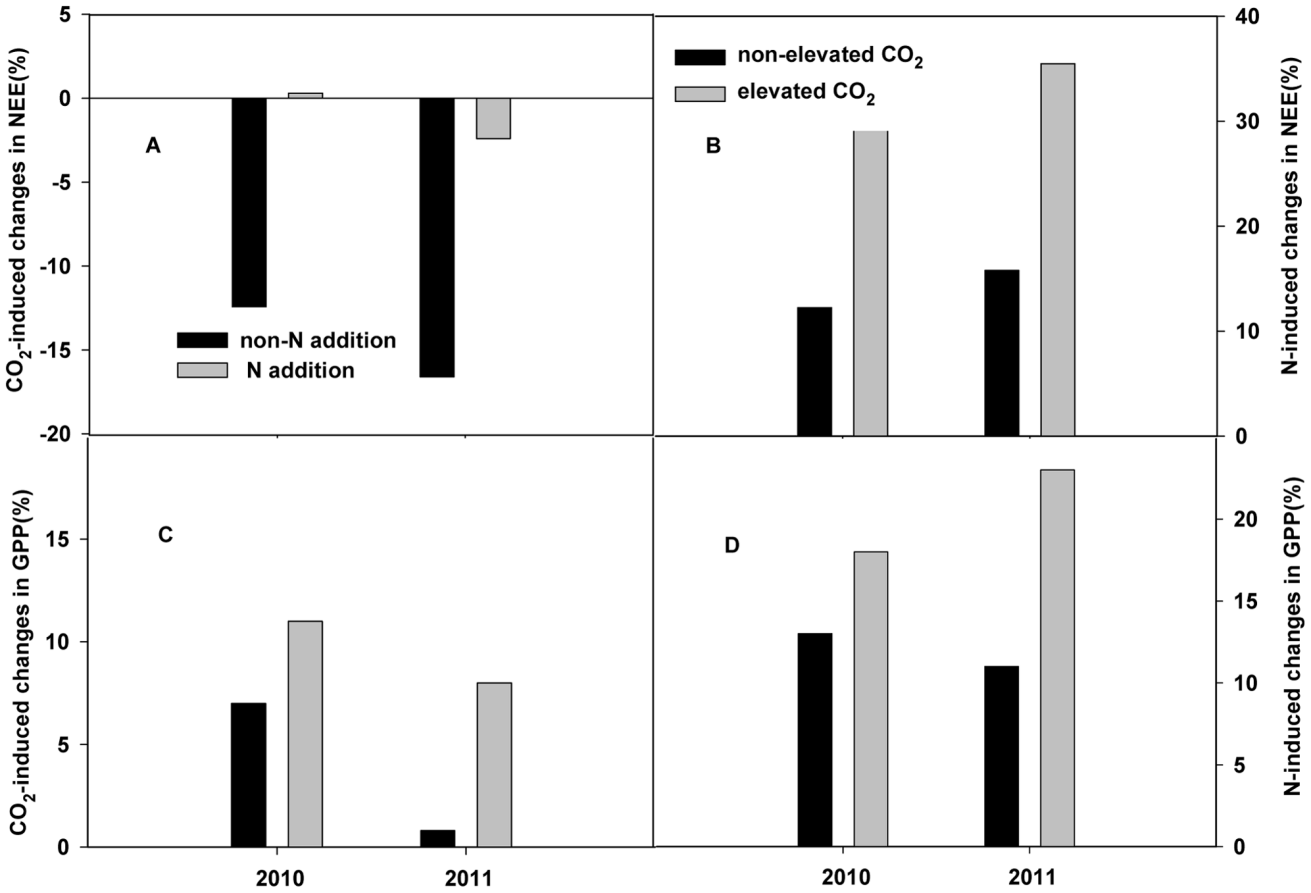
Elevated CO_2_-induced changes in NEE (A) and GPP (C) in the non-N-addition and N-addition plots, and the N-induced changes in NEE (B) and GPP (D) in the non-elevated CO_2_ and elevated CO_2_ plots in 2010 and 2011. See Figure 2 for abbreviations.

During the two years of the study, elevated CO_2_ significantly decreased NEE by 15% (P<0.01) measured by using RMANOVA. In addition, elevated CO_2_-induced decline in NEE was significantly lower in the enriched N plots than in ambient N plots. Elevated CO_2_ even slightly increased NEE in the enriched N plots in 2010 (Fig. 3A).

Unlike with NEE, elevated CO_2_ significantly enhanced ER and GPP by 20% and 4%, respectively (both P<0.01, Table 1, 2). Furthermore, there was a significant temporal shift in the ecosystem C fluxes over the entire period (P<0.05 for the CO_2_×D, Table 2). In addition, there were significant interactions between elevated CO_2_ and the years, which affected GPP and ER (P<0.05 for the CO_2_×year ). An increase in GPP, induced by elevated CO_2_ was higher in the enriched N plots than in the ambient N plots in both years (Fig. 3 C).

### Effects of N Addition on Ecosystem C Fluxes

Nitrogen addition significantly enhanced NEE, ER and GPP by 10%, 12% and 14%, respectively, (all P<0.01, Table 1, 2) during the two year study period. There was a significant temporal change in NEE, ER and GPP (P<0.001 for the N×D, Table 2) during the entire period. N-induced stimulation of seasonal average NEE and GPP was significantly higher in the elevated CO_2_ plots than that in the ambient CO_2_ plots in both years (Fig. 3B, D).

### Interactive Effects of Elevated CO_2_ and N Addition on Ecosystem C Fluxes

NEE was significantly affected by interactions between N addition and elevated CO_2_, but no interactive effects on ER and GPP were detected (Table 2). In addition, there was a significant temporal shift in NEE, ER and GPP by CO_2_ at ambient versus enriched N (P = 0.04, P = 0.002 and P = 0.052, respectively, for the CO_2_×N×D).

### Vegetation Cover and Leaf -Level Gas Exchange

**Figure 4:**
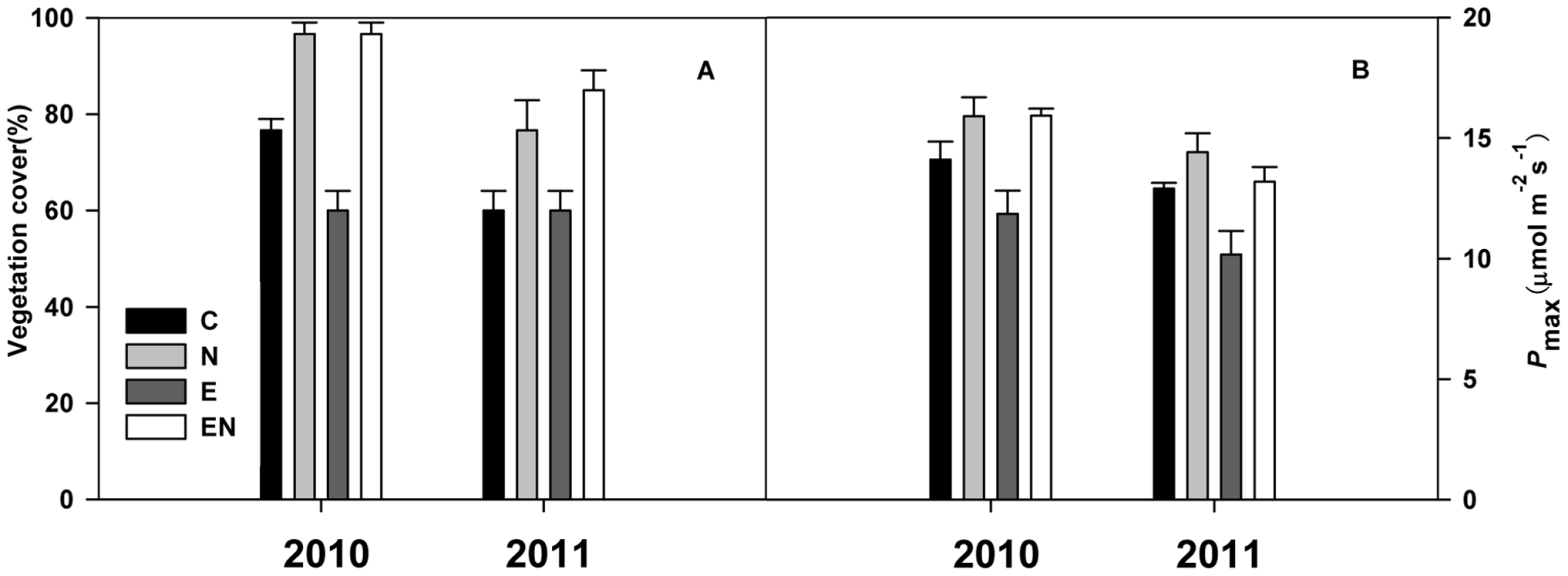
Figure 4. Responses of the vegetation cover (A) and the maximum photosynthetic rate (*P_max_*) of the dominant species *Calamagrostis angustifolia* (B) to elevated CO_2_ and N addition in July of 2010 and 2011. See Figure 2 for abbreviations.

In agreement with the elevated CO_2_ and N responses of NEE, elevated CO_2_ decreased the vegetation cover by 22% in 2010 (P<0.05) and reduced the maximum photosynthetic rate (P_max_) of the dominant species *C. angustifolia* by 16% in 2010 and 21% in 2011 (P<0.05). However, N addition enhanced vegetation cover by 26% in 2010 and 28% in 2011 (P<0.05) and increased P_max_ of *C. angustifolia* by 12% in 2010 and in 2011(P<0.05). Moreover, the vegetation cover and P_max_ of *C. angustifolia* were higher in 2010 than in 2011 (Fig. 4A, B).

### Controlling Factors of Ecosystem CO_2_ Fluxes

**Figure 5:**
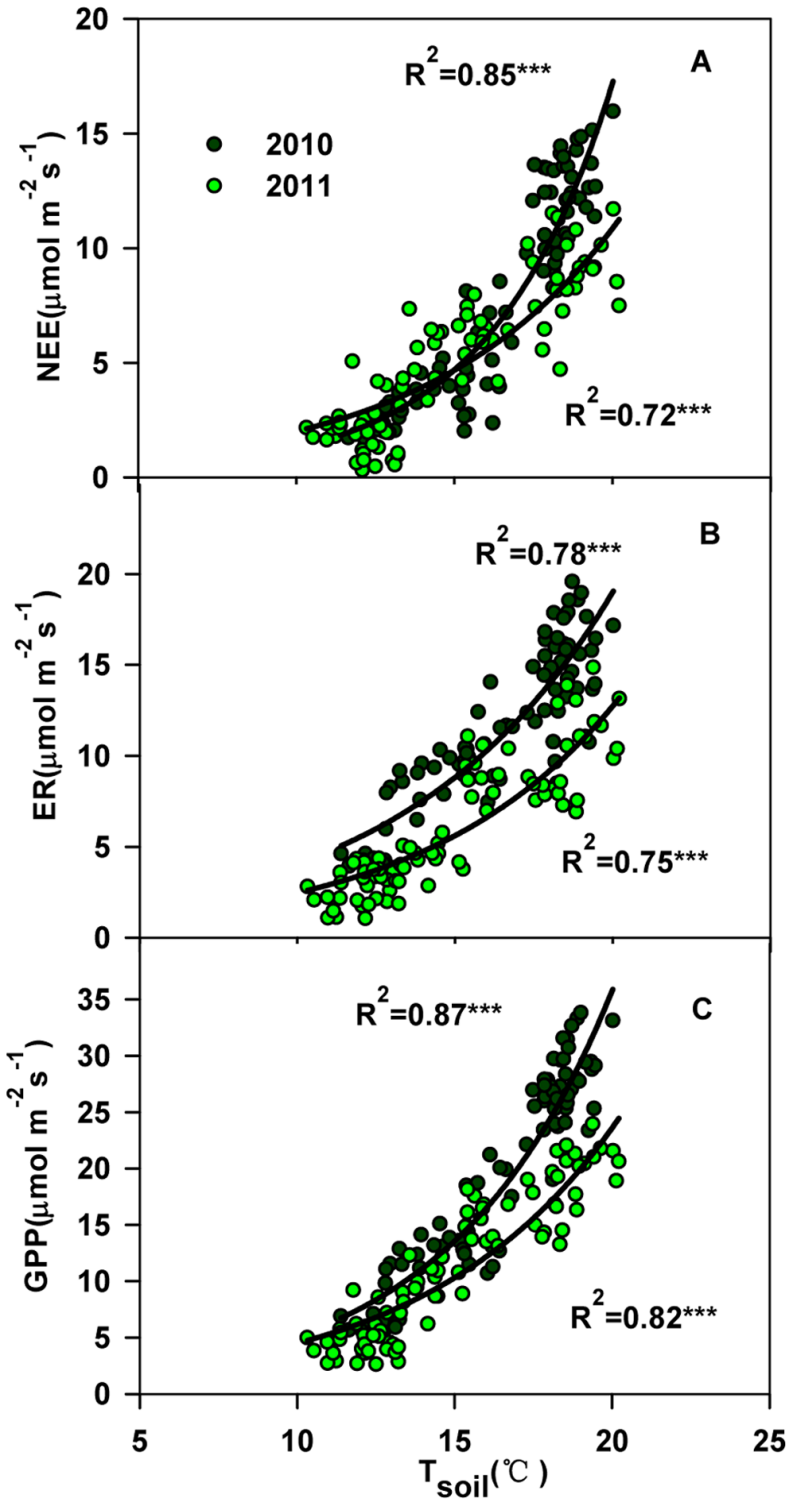
Temporal dependence of net ecosystem CO_2_ exchange (NEE), ecosystem respiration (ER) and gross primary productivity (GPP) to soil temperature (T_soil_, °C) across different plots in 2010 and 2011.

Exponential relationships between ecosystem C ﬂuxes and soil temperature (T_soil_) were found in both years (Fig. 5). T_soil_ explained 85%, 78% and 87% the variations in NEE, ER and GPP, respectively, in 2010. In 2011, soil temperature could explain 72%, 75% and 82% of the changes in NEE, ER and GPP, respectively. However, there was no significant relationship between ecosystem C fluxes and SWC (data not shown).

**Figure 6:**
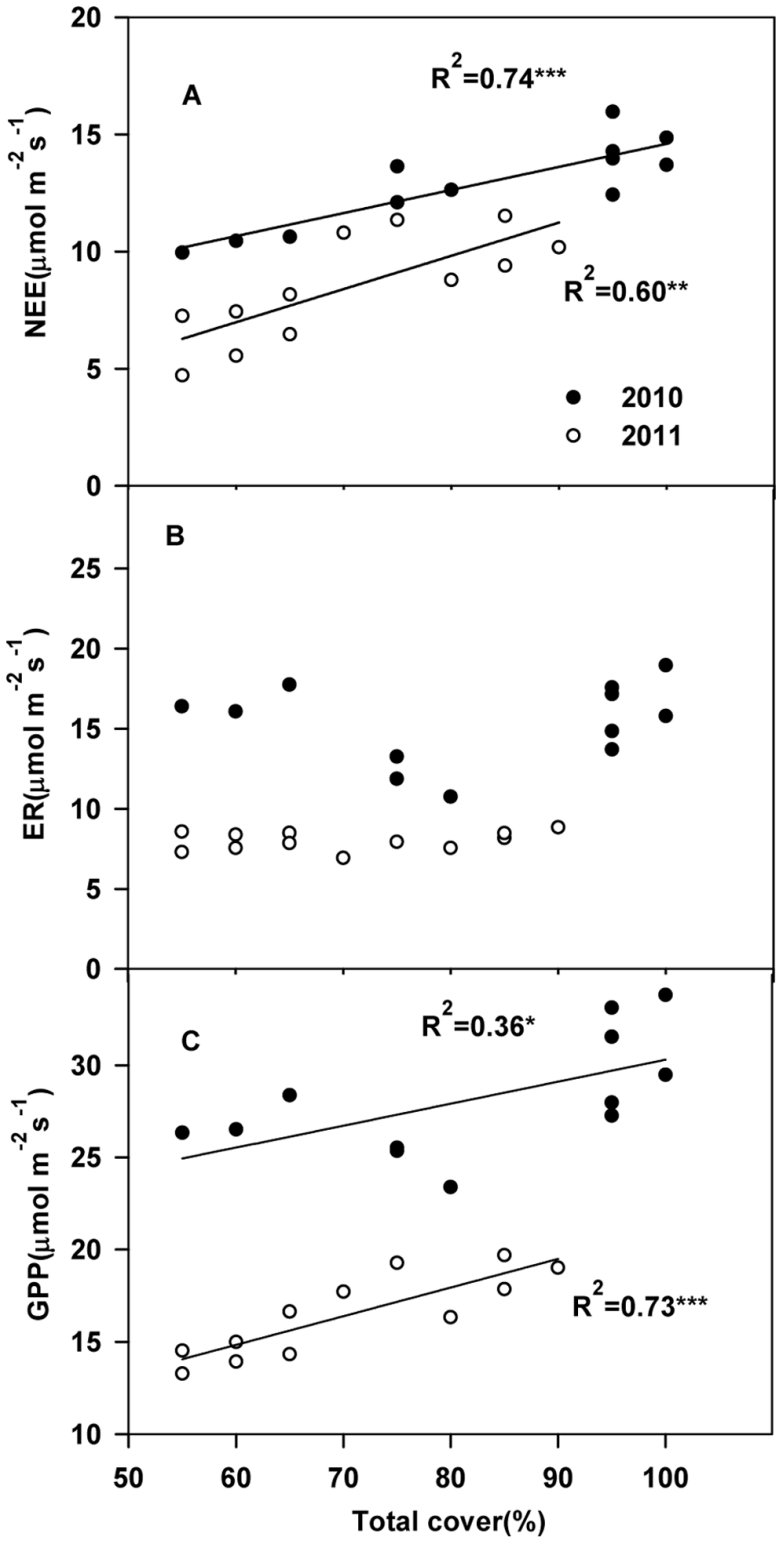
Correlations of net ecosystem CO_2_ exchange (NEE), ecosystem respiration (ER) and gross primary productivity (GPP) with vegetation cover across different plots in July of 2010 and 2011.

We found that both NEE (R^2^ = 0.74 and R^2^ = 0.60, P<0.01) and GPP (R^2^ = 0.36, P<0.01 and R^2^ = 0.73, P<0.01) were positively correlated with the plots’ vegetation cover. By contrast, there were no significant relationships between ER and vegetation cover in both years (Fig. 6B).

## Discussion

### Seasonal and Interannual Variability of Ecosystem C Fluxes

The seasonal dynamics of ecosystem carbon ﬂuxes observed in this study were high in mid-summer and low in the early and late growing seasons, which reﬂected air temperature (Fig. 1, 2). In addition, there were remarkable interannual variabilities in the ecosystem C fluxes (Fig. 2), which might be due to differences in air temperature in the early growing season. Specifically, the average air temperatures of May and of June were significantly lower in 2011 than in 2010. Consequently, a lower monthly NEE is expected in 2011, a hypothesis that would be consistent with Welp’s findings [26], who found that increases in spring air temperatures caused both GPP to increase by 74% and ER by 61% during the early part of the growing season (i.e., April, May, and June) in deciduous forest. The study by Zhou [27] indicated that in a lawn ecosystem, CO_2_ uptake significantly increased because of soil warming treatment. Moreover, in our study, ecosystem C fluxes were significantly exponentially related to soil temperature (Fig. 5). Less sensitive impact on ecosystem C fluxes to air temperature than soil temperature may be attributed to the wetland characteristics. In particular, the soil temperature is much lower than air temperature because the vegetation layer and the thick humus layer.

However, we found the C fluxes in 2010 are also higher than in 2011, when comparing fluxes recorded at the same soil temperature (Fig. 5), which implies that the vegetation cover partly accounted for the interannual differences in ecosystem C fluxes (Fig. 6).

In general, ecosystem C fluxes are closely related to SWC in grassland ecosystem [28], [29]. However, we found no significant relationship between ecosystem C fluxes and SWC in our freshwater wetland. A lack of SWC impact on ecosystem C fluxes may be due to the wetland soil and plant roots, which are water-saturated during the growing seasons, thus, the SWC ﬂuctuations have slight effects on ecosystem C fluxes. This was consistent with some previous findings [30], [31].

### Elevated CO_2_ Effects on Ecosystem CO_2_ Fluxes

Our study provides evidence that elevated CO_2_ can decrease NEE in this temperate wetland in northeast China because the changes in ER were more significant than changes in GPP for controlling the sign and magnitude of NEE (GPP and ER enhanced by 4% and 20%, respectively). This finding is consistent with a semi-arid grassland exposure to elevated CO_2_ [28]. In both years of our study, both CO_2_ treatments were net CO_2_ sinks, but exposure to elevated CO_2_ reduced CO_2_ sink strength by 15% over the two years. This negative effect of elevated CO_2_ on the net C uptake was in line with the trend observed in the Mojave Desert shrub ecosystem, which was exposed to elevated CO_2_ for eight years, resulting in a CO_2_ sink that was decreased by 30% [32]. The following two factors should, in theory, have affected the decline of NEE under elevated CO_2_ in our study.

1. Leaf photosynthetic capacity and vegetation cover. Elevated CO_2_ decreased both the vegetation cover that was fumigated for two months and the light-saturated photosynthesis of the leaves of the dominant species (*C. angustifolia*) (Fig. 4), which might be a reason for the decline in NEE. In addition, elevated CO_2_ decreased the chlorophyll concentration of *C. angustifolia* in cultivation experiments [33], which provided further evidence for the decline of leaf photosynthetic capacity. These results demonstrate that photosynthetic acclimation of leaf-level under elevated CO_2_ may simultaneously happen at the ecosystem scale.
2. Ecosystem C fluxes. In our study, despite the enhancd GPP response to elevated CO_2_ for most of experimental period (except for 23 June and 14 July 2011), elevated CO_2_ stimulated ER consistently to a greater extent compared to GPP during the experiment (Fig. 2, Table 1), resulting in reduced NEE, relative to control plots. Elevated CO_2_ led to higher ER and soil respiration (data unpublished), which might be explained by increased carbon inputs, an enhanced turnover rate in the roots, root hairs and SOM priming [5], [34]–[37]. Additionally, microbial biomass [38] may have also contributed to the higher respiration at elevated CO_2_ level.

Our findings indicated that increased ER under elevated CO_2_ casused lower NEE, compared to the control, which implies that increased root growth or activity could account for the much larger effect of elevated CO_2_ on ER. Although we could not confirm the exact source of the ecosystem respiration in this experiment, these results show that the potential for C sequestration will decreased to some extent in the next several decades at this study site.

### N Addition Effects on Ecosystem CO_2_ Fluxes

N addition significantly stimulated NEE in the two growing seasons in our ecosystem, largely due to N-induced increases in GPP, more so than those in ER (Table 1). The N-induced increases in canopy C uptake were partly caused by the stimulation of the leaf maximum photosynthetic rate of the dominant species (Fig. 4B), which was identical with another study [39], and by the higher vegetation cover on N addition plots (Fig. 4A). Stimulation in plant productivity provides more substrate for soil and plant respiration, leading to increases in ER under nitrogen fertilization. The positive effects have also been attributed to increased microbial biomass and activity [40] after fertilization. N stimulation of gross primary production on the Sanjiang Plain wetland (Fig. 2) is in accordance with meta-analysis results of positive N responses at global scales [9]. These results suggest that increased N deposition will enhance ecosystem carbon sequestration in the future.

### Interactive Effect of Elevated CO_2_ and N Addition on Ecosystem CO_2_ Fluxes

Some research has shown that elevated CO_2_ and N addition interact with each other in stimulating plant growth [18], [41]. Moreover, N addition has been reported to alleviate photosynthetic acclimation [42]. Thus, elevated CO_2_ and N addition might lead to potential interactive effects on ecosystem C fluxes. Oren [43] reported that soil fertility limits carbon sequestration in forest ecosystems with enriched CO_2_ level. Reich [44] found that a low availability of N progressively suppresses the positive response of plant biomass to elevated CO_2_ in perennial grassland. However, Aeschlimann [45] reported that there was a long-term stimulation in the net C assimilation by elevated CO_2_, but high N supply led to a lower net C input than low N supply in managed grassland.

At our study sites, a significant interactive effect between elevated CO_2_ and N addition on NEE (p = 0.007) was found during the experimental period (Table 2), and their combined effect increased NEE by 13% compared to that in the control plots (Table 1). The increased response of ecosystem CO_2_ fluxes to N addition under elevated CO_2_ compared to ambient CO_2_ (Fig. 3), together with the interactive effects of CO_2_ treatment and N treatment on NEE, indicate that elevated CO_2_ triggers physiological responses to N supply at the ecosystem scale.

In addition, there were significant interactions between the sampling date and experimental treatment on ecosystem C fluxes (CO_2_×D, N×D, and CO_2_×N×D, Table 2), which indicated that the effect of the experimental treatment on the ecosystem C fluxes altered over time. The mechanisms responsible for the changes in the response of ecosystem C fluxes to elevated CO_2_ and N addition over time may involve temporal heterogeneity in plant phenology, physiological activity, soil C and N mineralization, and root and soil organic matter decomposition and turnover [44].

We must note that there were low replication levels in our experiment. Moreover, our study was concerned with instantaneous C fluxes rather than cumulative C fluxes, which might prohibit comparison with other relevant studies. Even so, these results imply that a natural, unfertilized ecosystem experienced a decreased C sink than N-rich ecosystem at this site. Moreover, a severe N limitation in natural wetland may inhibit CO_2_ response to assimilation [46] and, consequently, to the ecosystem C balance. Thus, this study demonstrated that the net ecosystem CO_2_ uptake on the Sanjiang Plain wetland depends on adequate N supply.
